# Initial Phase Angle Is Independently Associated With Functional Independence at 6 Months After Stroke

**DOI:** 10.1111/ggi.70837

**Published:** 2026-09-10

**Authors:** Daisuke Ishiyama, Yoichiro Aoyagi, Miho Ohashi, Tatsuya Iwasawa, Tomonari Saito, Yuki Sakamoto, Junya Aoki, Kazumi Kimura

**Affiliations:** ^1^ Department of Rehabilitation Medicine Nippon Medical School Hospital Tokyo Japan; ^2^ Department of Neurology Nippon Medical School Hospital Tokyo Japan

**Keywords:** activities of daily living, phase angle, rehabilitation, sarcopenia, stroke

## Abstract

**Objectives:**

To examine the associations of initial sarcopenia and phase angle (PhA) with functional independence at 6 months after stroke onset.

**Methods:**

This observational study included 129 patients (median age, 74 years; 32.6% female) who experienced acute stroke and were admitted to the stroke unit of a tertiary care hospital between June 2022 and May 2023. The Barthel Index (BI) was assessed 6 months after stroke onset. Sarcopenia and PhA were evaluated during the acute phase. Multivariate logistic regression models were used to estimate the association between failure to achieve full independence (BI < 100) at 6 months and sarcopenia or PhA.

**Results:**

The incidence of failure to achieve full independence at 6 months was 27.1%, with a sarcopenia prevalence of 34.1%. The mean (standard deviation) PhA was 5.7 (1.0°) in males and 5.1 (0.9°) in females. After adjusting for age, sex, and NIHSS score, PhA was significantly associated with failure to achieve full independence at 6 months (odds ratio, 0.43; 95% confidence interval, 0.22–0.83; *p* = 0.012). In contrast, although sarcopenia showed a tendency toward association with the outcome, the association did not reach statistical significance (odds ratio, 2.66; 95% confidence interval, 0.95–7.46; *p* = 0.063).

**Conclusions:**

Initial PhA was independently associated with failure to achieve full independence at 6 months after stroke.

## Graphical abstract

Phase angle in the acute phase was independently associated with failure to achieve full independence at 6 months after stroke, whereas sarcopenia was not. Phase angle may serve as a practical indicator of integrated physiological characteristics related to functional recovery.

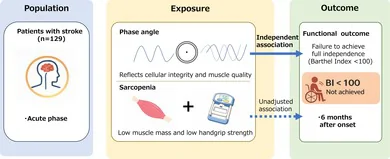

## 1 Introduction

Stroke is a leading cause of disability, and rehabilitation is a key component of care for patients with stroke‐related impairments. Approximately 25%–74% of stroke survivors experience some degree of disability [1], which imposes a substantial burden on patients, caregivers, and healthcare systems [2, 3, 4]. Early rehabilitation is essential for improving functional outcomes [5]; therefore, identifying factors associated with recovery during the acute phase of stroke is of particular importance.

Sarcopenia may substantially influence functional outcomes after stroke. It is characterized by reduced skeletal muscle mass and strength and has been associated with adverse outcomes, including increased disability, dysphagia, and mortality in patients with stroke [6, 7, 8]. The prevalence of sarcopenia in patients with stroke has been reported to range from approximately 15% to 60% depending on the disease phase [6, 7, 9, 10, 11, 12, 13]. Importantly, improvements in sarcopenia have been linked to better functional outcomes after stroke [14]. Therefore, assessing sarcopenia during the acute phase may be important for understanding and improving recovery trajectories. However, conventional assessments of sarcopenia may not fully capture muscle quality.

Phase angle (PhA), measured using bioelectrical impedance analysis, has emerged as a potential marker for predicting functional outcomes in patients with stroke. PhA reflects cellular integrity and muscle quality based on the relationship between resistance and reactance [15]. Lower PhA values are generally interpreted as indicating impaired cellular integrity, whereas higher values reflect a greater proportion of intact cells. Previous studies have demonstrated that PhA is associated with physical function and nutritional status, as well as functional recovery in patients with acute stroke [16, 17, 18, 19].

However, the associations of sarcopenia and PhA during the acute phase of stroke with long‐term functional outcomes remain unclear. Therefore, this study aimed to examine the associations of initial sarcopenia and PhA with functional independence at 6 months after stroke onset.

## 2 Methods

### 2.1 Study Design and Participants

This observational study included patients with acute stroke who were admitted to the stroke unit of Nippon Medical School Hospital (Bunkyo‐ku, Tokyo, Japan) between June 2022 and May 2023. Patients with cerebral infarction or hemorrhage were eligible. Exclusion criteria were as follows: lack of informed consent; pre‐onset modified Rankin Scale (mRS) [20] score ≥ 3; requirement for surgery; presence of metal implants; inability to measure handgrip strength due to impaired consciousness; in‐hospital death; and missing data. The study was conducted in accordance with the Declaration of Helsinki and was approved by the Institutional Review Board of Nippon Medical School Hospital (approval no. B‐2020‐302). Written informed consent was obtained from all participants or their families. This study was a post hoc analysis of a prospective registry.

### 2.2 Study Outcome

Functional outcomes were assessed using the Barthel Index (BI) [21]. The BI includes 10 items related to ADL, with the maximum score for each item indicating full independence in that activity. Each item has a minimum and maximum score: feeding (0–10), transfers (0–15), grooming (0–5), toilet use (0–10), bathing (0–5), mobility (0–15), stairs (0–10), dressing (0–10), bowels (0–10), and bladder (0–10). The total BI score ranges from 0 to 100 points. A follow‐up questionnaire, which included questions on the BI, was sent to the participants 6 months after stroke onset. To account for the marked ceiling effect of the BI (Figure S1), the primary outcome was dichotomized as full independence (BI = 100) versus non‐full independence (BI < 100). This cutoff was selected because a BI score of 100 represents complete independence in all basic activities of daily living, whereas a score < 100 indicates dependence in at least one BI item.

### 2.3 Bioelectrical Impedance Analysis

Bioelectrical impedance was measured using a multifrequency bioelectrical impedance analyzer (InBody S10; InBody Japan Inc., Tokyo, Japan). Measurements were performed in the supine position using an eight‐electrode system within 48 h of admission. The skeletal muscle mass index (SMI) and PhA were obtained from the measurements. SMI was calculated as appendicular muscle mass divided by height squared (kg/m^2^). PhA was calculated as follows:

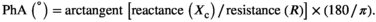




In this equation, reactance and resistance were measured at 50 kHz.

### 2.4 Sarcopenia Definition

Sarcopenia was defined according to the Asian Working Group for Sarcopenia (AWGS) 2019 criteria [22] as the presence of both low muscle mass and low muscle strength. Low SMI was defined as < 7.0 kg/m^2^ for men and < 5.7 kg/m^2^ for women, and low handgrip strength was defined as < 28 kg for men and < 18 kg for women. Handgrip strength was measured as the maximum value of two attempts using a Smedley hand dynamometer (TKK 5401, Takei Scientific Instruments Co. Ltd., Tokyo, Japan) on the nonparalyzed side within 48 h of admission, with the patient in the supine position.

### 2.5 Demographic and Clinical Characteristics

Demographic and clinical characteristics were collected, including age, sex, body mass index, pre‐onset mRS, admission diagnosis (cerebral infarction or hemorrhage), stroke severity, stroke history, comorbidities (hypertension, dyslipidemia, diabetes, atrial fibrillation, heart failure, and ischemic heart disease), length of hospital stay, and discharge destination (home or transfer). Stroke severity was assessed at admission using the National Institutes of Health Stroke Scale (NIHSS) [23].

### 2.6 Statistical Analysis

The normality of variables was assessed using the Shapiro–Wilk test. Between‐group comparisons were conducted using the unpaired *t*‐test, Mann–Whitney *U* test, or chi‐squared test, as appropriate. The independence rates for each BI item were compared between patients with and without sarcopenia using the chi‐squared test, and effect sizes were calculated using Cramer's *V*. To examine the associations with failure to achieve full independence (BI < 100) at 6 months, univariate and multivariate logistic regression analyses were performed. In the multivariate models, either sarcopenia or PhA was included as the main independent variable. Covariates were selected a priori based on their established prognostic importance for post‐stroke functional outcomes while considering the limited number of outcome events to minimize the risk of model overfitting. An exploratory multivariable logistic regression model including both sarcopenia and PhA was also performed to examine their interrelationship. Multicollinearity was assessed using variance inflation factors (VIF). As a supplementary analysis, linear regression models using the same covariates were conducted. Statistical significance was set at *p* < 0.05. Statistical analyses were performed using SPSS version 21 (IBM Corp., Armonk, NY, USA).

## 3 Results

**FIGURE 1:**
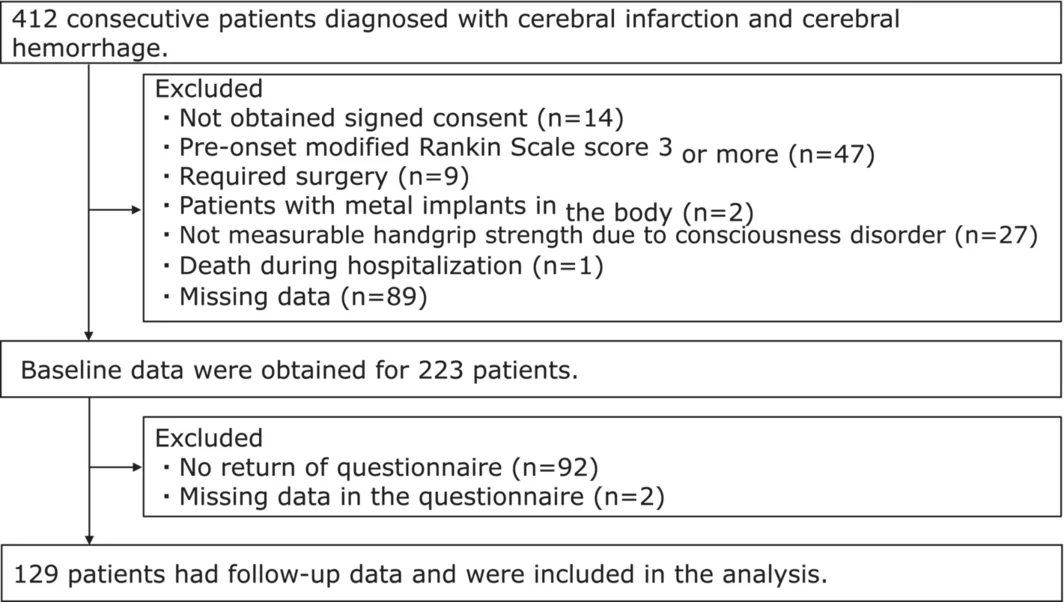
Flow diagram illustrating the patient selection process.

Of 412 eligible patients who met the inclusion criteria, 189 were excluded for the following reasons: no signed consent obtained (*n* = 14), pre‐onset mRS ≥ 3 (*n* = 47), required surgery (*n* = 9), presence of metal implants in the body (*n* = 2), inability to measure handgrip strength due to consciousness disorder (*n* = 27), death during hospitalization (*n* = 1), or missing data (*n* = 89). In addition, of the patients who received the questionnaire, 94 were excluded because they either did not return the questionnaire (*n* = 92) or did not fully complete it, resulting in missing data (*n* = 2). Therefore, 129 patients were included in the analysis (Figure 1).

The patient characteristics of the overall cohort and comparisons of variables according to the presence or absence of sarcopenia are shown in Table 1. The median (interquartile range) age of the patients was 74 (58–80) years, with 32.6% being female. The admission diagnoses for stroke were cerebral infarction (78.3%) and hemorrhage (21.7%). The median (interquartile range) BI score 6 months after stroke onset was 100 (93–100). The distribution of BI scores indicated a ceiling effect (Figure S1). Notably, 35 patients (27.1%) did not achieve full independence (BI < 100) at 6 months. Overall, 34.1% of all patients and 51.2% of patients aged 65 years or older had sarcopenia. The mean (standard deviation) PhA was 5.7 (1.0°) in males and 5.1 (0.9°) in females. Significant differences in age, BMI, pre‐onset mRS score, admission diagnosis, handgrip strength, SMI, PhA, and BI score at 6 months (*p* < 0.05) were observed between the sarcopenia and non‐sarcopenia groups.

**TABLE 1:** Patient characteristics.

	Overall (*n* = 129)	Sarcopenia (*n* = 44)	Non‐sarcopenia (*n* = 85)	*p*
Age, median (IQR)	74 (58–80)	79 (75–85)	64 (54–76)	< 0.001
Sex, *n* (%)				0.507
Male	87 (67.4%)	28 (63.6%)	59 (69.4%)	
Female	42 (32.6%)	16 (36.4%)	26 (30.6%)	
BMI (kg/m^2^), median (IQR)	23.0 (20.7–26.2)	21.5 (19.5–23.9)	23.9 (22.0–27.0)	< 0.001
Pre‐onset mRS score, *n* (%)				0.044
0	112 (86.8%)	35 (79.5%)	77 (90.6%)	
1	10 (7.8%)	7 (15.9%)	3 (3.5%)	
2	7 (5.4%)	2 (4.5%)	5 (5.9%)	
Admission diagnosis, *n* (%)				0.003
Cerebral infarction	101 (78.3%)	41 (93.2%)	60 (70.6%)	
Cerebral hemorrhage	28 (21.7%)	3 (6.8%)	25 (29.4%)	
NIHSS score at admission, median (IQR)	3 (1–7)	3 (1–5)	4 (1–7)	0.347
Stroke history, *n* (%)				0.103
First stroke	103 (80.6%)	32 (72.7%)	72 (84.7%)	
Recurrent stroke	25 (19.4%)	12 (27.3%)	13 (15.3%)	
Comorbidity, *n* (%)				
Hypertension	85 (65.9%)	32 (72.7%)	53 (62.4%)	0.239
Dyslipidemia	49 (38.0%)	20 (45.5%)	29 (34.1%)	0.208
Diabetes	23 (18.6%)	9 (20.5%)	15 (17.6%)	0.698
Atrial fibrillation	13 (10.1%)	4 (9.1%)	9 (10.6%)	0.789
Heart failure	4 (3.1%)	1 (2.3%)	3 (3.5%)	0.696
Ischemic heart disease	7 (5.4%)	1 (2.3%)	6 (7.1%)	0.255
Handgrip strength, mean (SD)	24.1 (10.1)	17.3 (6.7)	27.7 (9.7)	< 0.001
SMI [kg/m^2^], mean (SD)	6.8 (1.3)	5.8 (0.9)	7.4 (1.1)	< 0.001
Phase angle [degree], mean (SD)	5.5 (1.0)	4.8 (0.8)	5.9 (0.9)	< 0.001
Length of hospitalization [days], median (IQR)	17 (12–21)	16 (12–21)	17 (12–23)	0.482
Discharge destination, *n* (%)				0.585
Home	60 (46.5%)	19 (43.2%)	41 (48.2%)	
Transfer	69 (53.5%)	25 (56.8%)	44 (51.8%)	
BI score at 6 months, median (IQR)	100 (93–100)	100 (80–100)	100 (100–100)	0.001

Abbreviations: BI, Barthel Index; BMI, body mass index; IQR, interquartile range; mRS, modified Rankin Scale; NIHSS, National Institutes of Health Stroke Scale; SD, standard deviation; SMI, skeletal muscle mass index.

**FIGURE 2:**
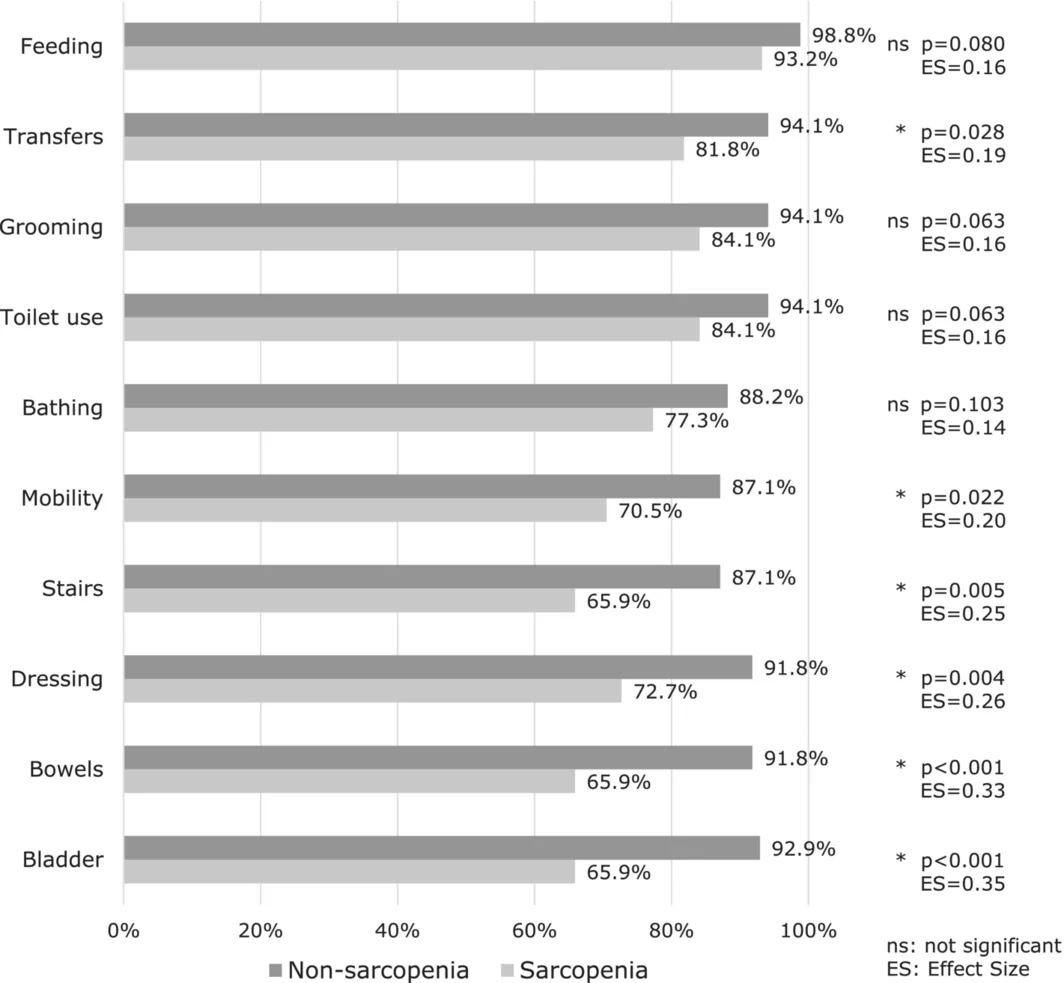
The independence rate of each BI item according to the presence or absence of sarcopenia.

The independence rates for each BI item based on the presence or absence of sarcopenia are shown in Figure 2. Significant differences in the independence rates (non‐sarcopenia vs. sarcopenia) were observed for transfers (94.1% vs. 81.8%), mobility (87.1% vs. 70.5%), stairs (87.1% vs. 65.9%), dressing (91.8% vs. 72.7%), bowel (91.8% vs. 65.9%), and bladder (92.9% vs. 65.9%) (*p* < 0.05). These findings are descriptive in nature.

The results of the univariate and multivariate logistic regression analyses are presented in Table 2. Univariate logistic regression analyses showed that both sarcopenia and PhA were associated with failure to achieve full independence at 6 months after stroke onset. Other variables associated with failure to achieve full independence following univariate analyses included age, BMI, pre‐onset mRS score, NIHSS score at admission, and stroke history (*p* < 0.05). After adjustment for age, sex, and NIHSS score, multivariate logistic regression analysis showed that PhA remained significantly associated with failure to achieve full independence at 6 months (odds ratio, 0.43; 95% confidence interval, 0.22–0.83; *p* = 0.012). In contrast, although sarcopenia tended to be associated with the outcome, the association did not reach statistical significance (odds ratio, 2.66; 95% confidence interval, 0.95–7.46; *p* = 0.063).

**TABLE 2:** Logistic regression models of the associations between failure to achieve full independence (BI < 100) at 6 months after stroke onset and sarcopenia or phase angle.

	Univariate		Multivariate (sarcopenia model)		Multivariate (phase angle model)	
	OR (95% CI)	*p*	OR (95% CI)	*p*	OR (95% CI)	*p*
Sarcopenia	3.89 (1.72–8.78)	0.001	2.66 (0.95–7.46)	0.063		
Phase angle	0.36 (0.22–0.59)	< 0.001			0.43 (0.22–0.83)	0.012
Age	1.08 (1.04–1.13)	< 0.001	1.06 (1.01–1.12)	0.011	1.04 (0.99–1.10)	0.091
Sex (male)	0.45 (0.20–1.02)	0.054	0.51 (0.20–1.31)	0.161	0.72 (0.28–1.87)	0.496
BMI (per kg/m^2^)	0.88 (0.79–0.98)	0.019				
Pre‐onset mRS						
0	(Reference)					
1	4.96 (1.30–18.93)	0.019				
2	2.48 (0.52–11.80)	0.254				
Admission diagnosis (cerebral hemorrhage)	1.10 (0.43–2.78)	0.847				
NIHSS score (per point)	1.10 (1.03–1.18)	0.005	1.15 (1.05–1.25)	0.002	1.15 (1.05–1.26)	0.002
Stroke history (recurrent stroke)	2.62 (1.05–6.52)	0.039				
Hypertension	1.42 (0.61–3.30)	0.419				
Dyslipidemia	1.12 (0.51–2.49)	0.774				
Diabetes	1.44 (0.56–3.75)	0.450				
Atrial fibrillation	0.79 (0.20–3.05)	0.729				
Heart failure	Not estimable (no events)	—				
Ischemic heart disease	3.91 (0.83–18.47)	0.098				

Abbreviations: BI, Barthel Index; BMI, body mass index; CI, confidence interval; mRS, modified Rankin Scale; NIHSS, National Institutes of Health Stroke Scale; OR, odds ratio.

In an exploratory multivariable logistic regression model including both sarcopenia and PhA (Table S1), only PhA remained significantly associated with the outcome. Multicollinearity was acceptable, with VIFs < 3 for all variables.

In the supplementary linear regression analyses (Table S2), both sarcopenia and PhA were associated with the BI score at 6 months after stroke onset (*p* < 0.05), with standardized *β* coefficients of −0.268 for sarcopenia and 0.484 for PhA; however, these findings should be interpreted with caution due to potential violations of model assumptions, including the ceiling effect of the BI.

## 4 Discussion

The main finding of this study is that both sarcopenia and PhA were associated with failure to achieve full independence at 6 months after stroke in univariate analyses; however, after adjustment for confounding factors, only PhA remained independently associated with the outcome. These findings suggest that the physiological characteristics reflected by PhA may play a role in functional recovery after stroke.

In the present study, both sarcopenia and PhA were associated with functional independence in the univariate analyses, consistent with previous reports. Although sarcopenia and PhA are related measures, they provide different physiological information. Whereas sarcopenia reflects impaired muscle mass, strength, and physical performance, PhA has been proposed as a surrogate marker of muscle quality but also reflects hydration status, nutritional status, inflammation, and overall cellular integrity [24, 25, 26, 27]. Notably, only PhA remained independently associated after adjustment for confounding factors. This finding suggests that PhA may capture integrated physiological characteristics relevant to functional recovery after stroke, rather than muscle quality alone.

When both sarcopenia and PhA were included in the same model, only PhA remained significantly associated with the outcome. This finding suggests that these variables may reflect overlapping aspects of skeletal muscle status. However, given this conceptual overlap [28, 29], the results of this analysis should be interpreted as exploratory.

PhA has several practical advantages in clinical settings. It can be measured in a static position, even in patients with limited mobility, and can be obtained objectively with minimal dependence on patient effort. In addition, PhA is derived from resistance and reactance, providing a physiologically interpretable parameter. These characteristics may enhance its utility as a complementary indicator in the assessment of skeletal muscle condition.

When comparing ADL sub‐items, lower independence rates were observed in several items among patients with sarcopenia, including transfers, mobility, stairs, dressing, bowel, and bladder. These activities have been reported to be associated with muscle strength and physical function, which are key components of sarcopenia [30, 31, 32]. In addition, previous studies have suggested associations between sarcopenia and bowel and bladder function [33, 34]. However, these findings should be considered descriptive and interpreted with caution, as the present study was not designed to examine item‐level associations in detail. Further studies with larger sample sizes are needed to clarify these relationships.

This study has several limitations. First, it was conducted at a single center, which may limit the generalizability of the findings. Second, missing baseline and follow‐up data may have introduced selection and responder bias. Although baseline characteristics did not differ significantly between included and excluded participants (Table S3), loss to follow‐up may have affected the representativeness of the final analytical cohort and, consequently, the estimated association between PhA and functional outcome. In addition, although the multivariable models were adjusted for age, sex, and NIHSS score, residual confounding from other clinically relevant variables not included in the multivariable models cannot be excluded. Therefore, as the final analytical cohort predominantly consisted of patients with mild stroke, with a median NIHSS score of 3 (IQR, 1–7), the present findings should be interpreted with caution, and their generalizability to patients with moderate or severe stroke may be limited. Third, sarcopenia could not be assessed in some patients due to missing grip strength and body composition data. Fourth, PhA may be influenced by hydration status and fluid distribution [35, 36]. In this study, most patients (125 of 129, 96.9%) received intravenous fluids at the time of measurement; however, detailed information on fluid volume, edema, or fluid balance was not available, and residual confounding cannot be excluded. Fifth, PhA was assessed as a whole‐body measure, and the potential impact of segmental PhA remains unclear, particularly given the asymmetrical muscle changes that can occur after stroke. Finally, although we dichotomized the BI at a score of 100 because this threshold represents complete independence in basic activities of daily living [21] and addressed the marked ceiling effect observed in our cohort (Figure S1), previous studies have also highlighted the ceiling effect and analytical limitations of the BI, particularly in patients with mild stroke [4, 37, 38]. Therefore, alternative thresholds or ordinal modeling approaches may also be appropriate, and the present findings should be interpreted within the context of this methodological choice. Future studies including larger cohorts with a broader spectrum of stroke severity are warranted to improve the generalizability of these findings and to determine whether the present results are consistent across alternative BI thresholds or ordinal analytical approaches.

## 5 Conclusion

Initial PhA was independently associated with failure to achieve full independence at 6 months after stroke, whereas sarcopenia was not independently associated with the outcome. These findings suggest that assessment of integrated physiological characteristics, including skeletal muscle quality, cellular integrity, and nutritional status, during the acute phase may be useful for identifying patients at risk of poor functional recovery.

## Funding

This work was supported by JSPS KAKENHI, JP21K21262.

## Conflicts of Interest

The authors declare no conflicts of interest.

## Supporting information


**Table S1:** Exploratory multivariable analysis of sarcopenia and phase angle.
**Table S2:** Linear regression models of the relationships among BI at 6 months after onset and sarcopenia or phase angle.
**Table S3:** Comparisons of patient characteristics between obtained and missing follow‐up data.

**Figure S1:** Distribution of the Barthel Index score at 6 months after stroke onset. (A) Overall distribution. (B) Distribution according to sarcopenia status.

## Data Availability

The data that support the findings of this study are available from the corresponding author upon reasonable request.
