# Supplementary material for: Initial Phase Angle Is Independently Associated With Functional Independence at 6 Months After Stroke
Source: Geriatr Gerontol Int. 2026 Sep 10;26(9):e70837. doi: 10.1111/ggi.70837 (PMC13561573; doi:10.1111/ggi.70837)
Supplement: Supplementary file 1 [file GGI-26-0-s002.docx]

| Supplementary Table 1. Exploratory multivariable analysis of sarcopenia and phase angle | | | |
| --- | --- | --- | --- |
|  | Multivariate (simultaneous model) | |  |
|  | OR (95%CI) | *P* value | VIF |
| Sarcopenia | 1.91 (0.64–5.71) | 0.249 | 1.54 |
| Phase angle | 0.48 (0.24–0.96) | 0.039 | 2.07 |
| Age | 1.04 (0.98–1.09) | 0.187 | 1.86 |
| Sex (male) | 0.66 (0.25–1.75) | 0.404 | 1.13 |
| NIHSS score (per point) | 1.16 (1.06–1.26) | 0.001 | 1.02 |
| OR, odds ratio; CI, confidence interval; NIHSS, National Institutes of Health Stroke Scale | | | |

| Supplementary Table 2. Linear regression models of the relationships among BI at six months after onset and sarcopenia or phase angle | | | | | | | | | | | |
| --- | --- | --- | --- | --- | --- | --- | --- | --- | --- | --- | --- |
|  | Univariate |  |  |  | Multivariate (sarcopenia model) | |  |  | Multivariate (phase angle model) | |  |
|  | B (95%CI) | β | *P* value |  | B (95%CI) | β | *P* value |  | B (95%CI) | β | *P* value |
| Sarcopenia | -13.25 (-21.09 to -5.40) | -0.284 | 0.001 |  | -12.52 (-21.39 to -3.64) | -0.268 | 0.006 |  |  |  |  |
| Phase angle | 9.47 (5.97 to 12.96) | 0.429 | <0.001 |  |  |  |  |  | 10.66 (6.02 to 15.31) | 0.484 | <0.001 |
| Age | -0.36 (-0.64 to -0.08) | -0.217 | 0.014 |  | -0.09 (-0.40 to 0.23) | -0.054 | 0.578 |  | 0.17 (-0.17 to 0.50) | 0.101 | 0.322 |
| Sex (male) | 10.33 (2.25 to 18.41) | 0.219 | 0.013 |  | 9.45 (1.78 to 17.12) | 0.200 | 0.016 |  | 4.13 (-3.53 to 11.79) | 0.088 | 0.288 |
| BMI (per kg/m^2^) | 0.95 (0.09 to 1.80) | 0.190 | 0.030 |  |  |  |  |  |  |  |  |
| Pre-onset mRS |  |  |  |  |  |  |  |  |  |  |  |
| 0 | (Reference) |  |  |  |  |  |  |  |  |  |  |
| 1 | -23.21 (-37.12 to -9.31) | -0.281 | 0.001 |  |  |  |  |  |  |  |  |
| 2 | -13.93 (-30.34 to 2.49) | -0.143 | 0.096 |  |  |  |  |  |  |  |  |
| Admission diagnosis (cerebral hemorrhage) | -2.21 (-11.62 to 7.20) | -0.041 | 0.643 |  |  |  |  |  |  |  |  |
| NIHSS score (per point) | -0.73 (-1.37 to -0.09) | -0.198 | 0.025 |  | -0.85 (-1.45 to -0.25) | -0.230 | 0.006 |  | -0.86 (-1.43 to -0.29) | -0.232 | 0.004 |
| Stroke history (recurrent stroke) | -13.22 (-22.76 to -3.68) | -0.236 | 0.007 |  |  |  |  |  |  |  |  |
| Hypertension | -0.73 (-8.91 to 7.46) | -0.016 | 0.861 |  |  |  |  |  |  |  |  |
| Dyslipidemia | 0.42 (-7.58 to 8.41) | 0.009 | 0.918 |  |  |  |  |  |  |  |  |
| Diabetes | -0.30 (-10.27 to 9.68) | -0.005 | 0.824 |  |  |  |  |  |  |  |  |
| Atrial fibrillation | 3.54 (-9.33 to 16.42) | 0.048 | 0.587 |  |  |  |  |  |  |  |  |
| Heart failure | 9.64 (-12.69 to 31.97) | 0.076 | 0.394 |  |  |  |  |  |  |  |  |
| Ischemic heart disease | -14.29 (-31.24 to 2.66) | -0.146 | 0.098 |  |  |  |  |  |  |  |  |
| Adjusted R^2^ |  |  |  |  | 0.151 |  |  |  | 0.226 |  |  |
| BI, Barthel Index; CI, confidence interval; BMI, body mass index; mRS, modified Rankin Scale; NIHSS, National Institutes of Health Stroke Scale | | | | | | | | | | | |

| Supplementary Table 3. Comparisons of patient characteristics between obtained and missing follow-up data. | | | |
| --- | --- | --- | --- |
|  | Obtained (n=129) | Missing (n=94) | *P* value |
|  |  |  |  |
| Age, median (IQR) | 74 (58–80) | 72 (63-81) | 0.725 |
| Sex, n (%) |  |  | 0.787 |
| Male | 87 (67.4%) | 65 (69.1%) |  |
| Female | 42 (32.6%) | 29 (30.9%) |  |
| BMI [kg/m^2^], median (IQR) | 23.0 (20.7–26.2) | 23.4 (20.7–26.4) | 0.946 |
| Pre-onset mRS score, n (%) |  |  | 0.223 |
| 0 | 112 (86.8%) | 85 (90.4%) |  |
| 1 | 10 (7.8%) | 7 (8.5%) |  |
| 2 | 7 (5.4%) | 1 (1.1%) |  |
| Admission diagnosis, n (%) |  |  | 0.134 |
| Cerebral Infarction | 101 (78.3%) | 81 (86.2%) |  |
| Cerebral hemorrhage | 28 (21.7%) | 13 (13.8%) |  |
| NIHSS score at admission, median (IQR) | 3 (1–7) | 4 (1–8) | 0.349 |
| Stroke history, n (%) |  |  | 0.384 |
| First stroke | 103 (80.6%) | 80 (85.1%) |  |
| Recurrent stroke | 25 (19.4%) | 14 (14.9%) |  |
| Comorbidity, n (%) |  |  |  |
| Hypertension | 85 (65.9%) | 64 (68.1%) | 0.731 |
| Dyslipidemia | 49 (38.0%) | 29 (30.9%) | 0.270 |
| Diabetes | 23 (18.6%) | 19 (20.2%) | 0.764 |
| Atrial fibrillation | 13 (10.1%) | 13 (13.8%) | 0.389 |
| Heart failure | 4 (3.1%) | 3 (3.2%) | 0.958 |
| Ischemic heart disease | 7 (5.4%) | 5 (5.3%) | 0.972 |
| Handgrip strength, mean (SD) | 24.1 (10.1) | 22.1 (10.9) | 0.157 |
| SMI [kg/m^2^], mean (SD) | 6.8 (1.3) | 6.7 (1.3) | 0.623 |
| Sarcopenia, n (%) | 44 (34.1%) | 33 (35.1) | 0.877 |
| Phase angle [degree], mean (SD) | 5.5 (1.0) | 5.3 (1.0) | 0.166 |
| Length of hospitalization [day], median (IQR) | 17 (12–21) | 15 (12–22) | 0.805 |
| Discharge destination, n (%) |  |  | 0.607 |
| Home | 60 (46.5%) | 42 (50.0%) |  |
| Transfer | 69 (53.5%) | 42 (50.0%) |  |
|  |  |  |  |
| IQR, interquartile range; SD, standard deviation; BMI, body mass index; mRS, modified Rankin Scale; NIHSS, National Institutes of Health Stroke Scale; SMI, skeletal muscle mass index, BI. | | | |
